# Open access and open source in chemistry

**DOI:** 10.1186/1752-153X-1-3

**Published:** 2007-02-19

**Authors:** Matthew H Todd

**Affiliations:** 1 School of Chemistry, University of Sydney, NSW 2006, Sydney, Australia.

## Abstract

Scientific data are being generated and shared at ever-increasing rates. Two new mechanisms for doing this have developed: open access publishing and open source research. We discuss both, with recent examples, highlighting the differences between the two, and the strengths of both.

## Background

The internet continues to transform the way we do science. We can search very large digital repositories of literature effectively with great speed. We can disseminate our data effectively instantaneously by posting it on the web. We can argue and collaborate with communication tools that overcome the most serious physical obstacles. In this article we look at two broad themes of this change, open access and open source. This commentary marks a very significant development in open access chemistry publishing, the launch of the Chemistry Central Journal.

One of the most significant impacts of the web is that it is essentially a structure that has emerged 'free of charge.' We pay for access, but seldom, as users, pay directly for the infrastructure. One of the world's most powerful computer systems is that operated by Google for managing its web searches [1]. Many of us use this tool daily to find information that ranges from the trivial (structure of a chemical, website URL of a colleague) to the more advanced (undergraduate teaching resources, commercial relevance of compounds). Yet the search engine, and the enormous infrastructure required to run it, is financed by advertising: we pay no access or subscription charges. Further, tools that are being developed as offshoots of the engine are also funded by this mechanism. One can download a desktop search tool free of charge that rapidly indexes all locally-saved PDF files, allowing us to search hundreds of relevant papers for the occurrence of particular chemical terms.

The web is growing, as is the speed with which we can move around it. (The driving forces behind this are probably not academic scientific research). This has resulted in two significant new developments in the way we carry out formal scientific research. One is a mechanism of distributed collaboration called open source research. The other is a new way of publishing peer-reviewed research, known as open access.

## Open Access

The term open access has come to mean data (usually peer-reviewed journal articles) that may be read free of charge. Rigorous peer-review, journal management and journal production costs are significant, and traditional scholarly publication has typically raised this money *via *subscription. Such costs can be modified depending on the point of access, for example the archives of many chemical journals are free to access from the world's least developed countries *via *the Programme for the Enhancement of Research Information [2], run by a charitable arm of the International Council for Science, a non-governmental organisation [3]. The core mechanism of funding the publications *via *subscription, however, remains the same.

Scientific publishing has been one of the drivers in the field of open access. There is some debate about how best to fund open access publishing [4], but the experiment is well underway. The history of open access has been well documented [5]. The preprint server arXiv has been running since 1991, and now accepts papers beyond the initial remit of high energy physics [6]. There are currently nearly 400,000 papers on the site. BioMed Central originally began peer-reviewed open access publishing in 2000 [7]. In 2003, Public Library of Science Biology was launched, and has been followed by eight further journals. BioMed Central now publishes over 100 independent titles. In Chemistry, there are currently over 50 open access journals [8]. Arkivoc has been publishing synthetic organic chemistry papers since 2000 [9], and the Beilstein Journal of Organic Chemistry commenced in August 2005 [10]. These journals are both successful as academic enterprises (PLoS Biology's impact factor is already around 14). Moreover, funding agencies such as the Howard Hughes Medical Institute in the US and the Wellcome Trust in the UK are requiring their investigators to deposit articles arising from their funding in open access databases such as PubMed Central within 6-12 months of publication [11].

Open access is not confined to journal publication. Open access online search tools are making it easier to find important information for chemical research. Directories of known molecules such as SciFinder and Beilstein are still subscription-only services, but it is possible to search for commercially-available compounds *via *a free online engine [12]. Searches may be performed on structures (not just text strings). At the time of writing the database contains 5.6 million compounds (Personal communication, Klaus Gubernator, CEO, emolecules) [13]. PubChem is a freely accessible database of millions of compounds maintained by the National Institutes of Health that may be searched for, amongst other things, biological activity of small molecules [14]. As with the database of commercially-available compounds, the search may be done with structures rather than text. A commercially-based enterprise, Collaborative Drug Discovery, is also taking diverse sets of biological data and making them highly searchable for the relevant groups involved in research into various parasitic diseases [15]. Partners in this venture include the Sandler Center which hosts an open database of compounds, screens and protocols for various parasitic organisms [16].

The rapid pace of development in open access in general means we cannot hope to be comprehensive here. Two significant recent developments include Google's Book Project [17] and the Open Content Alliance [18], but for further general discussion on open access and more recent developments, the reader is directed to a comprehensive resource [19].

## Open Source

Open source refers to any enterprise where data (e.g. journal article, piece of software) may be modified by the relevant community and those modifications may be recontributed to the larger whole. There is therefore a very significant distinction between this and open access: open source data are mutable.

What is the advantage of such an enterprise? An open source biomedical research community that started in 2005, the Synaptic Leap (see below), has as its motto the quote "None of us is as smart as all of us" (This quote has an uncertain attribution. Some sources credit Robert Oppenheimer, some that it is a Japanese Proverb). The promise of open source lies in the massively collaborative efforts that may be undertaken, efforts that are effective only through the increased speed and scale of communication *via *the web. Stereotypically these contributors are unpaid volunteers, but a major survey of hacker activity found that 30% of those taking part in computer science open source projects were paid [20]. Regardless, open source functions through the actions of many contributors from diverse backgrounds. There are two consequences of this. First, peer-review of the traditional kind (fixed duration, pre-publication) is not present - the peer review in open source is gradual and post-publication. Second, academic open source contributions tend to be of a higher quality and/or honesty than a cynic may suppose, a phenomenon known as the "gift relationship" [21].

Open source has delivered significant successes in recent years, and almost as much controversy. The number of people using Wikipedia may just be larger than the number who deny its usefulness. Wikipedia recently fared quite well in a head-to-head against the Encyclopaedia Britannica, but it is inevitable that such an enterprise contains errors (The original comparison was carried out by Nature, and a discussion of the ongoing argument between them and Britannica may be viewed on the Nature website) [22]. As with all open source projects, the final product emerges gradually through a large number of iterative changes. In amongst reports of the thousands of spurious edits of the page for US presidents, it is worthwhile remembering that Wikipedia currently contains over five million articles, in 250 languages (1.5 million in English), has emerged within the last six years and is available free of charge. Britannica has been published since 1768 and contains approximately 120,000 articles in English in the online edition, and operates on a subscriber model. It is also worth remembering that the Oxford English Dictionary, when it was being originally compiled, relied on contributions from volunteers, including the notorious William Minor of the asylum at Broadmoor [23]. Open source successes in computer science, such as Linux and Firefox, have been far less controversial, and have delivered high-quality products competing with those from major software firms.

Open source is also very active in Chemistry, though knowledge of these promising contributions is not widespread [24]. Examples may be categorised as informal communities, chemical tools and collaborative research groups.

### 1) Informal communities

Blogs (web sites hosted by individuals, where readers may post comments) are informal environments where science can be discussed. Such sites will continue to multiply. While blogs have a reputation as not being serious science, useful scientific contributions do emerge. As an example, various experimental procedures have been described on long-running chemistry blogs Tenderbutton [25] and Org Prep Daily [26]. Details of experimental procedures are described, along with extra content such as pictures of crystals from the experiments. User comments describe improvements and modifications. Anecdotal discussions such as these can only be useful to empirical scientists. That the web is so searchable means these discussions may easily be found. If a chemist has a problem with a reaction, they will typically ask their colleagues in the same group/lab/building for advice. Open source communities do exactly the same thing, but over much larger (geographical and social) distances.

### 2) Chemical tools

Several proprietary drawing packages are widely used, but open source alternatives exist. For example, free chemical drawing tools that are in development include Bkchem [27], and JchemPaint [28]. Sophisticated tools exist for viewing molecules in three dimensions conveniently in web pages, such as Jmol [29]. A related product, MDLChime, is free to use but not open source [30]. A directory of open source chemistry projects may be found at the Open Science Project [31]. The Blue Obelisk movement seeks to ensure interoperability in these applications by maintaining a set of open standards and, amongst other things, maintaining a list of algorithm specifications in chemoinformatics [32,33]. Related tools of relevance to drug design are discussed elsewhere [34,35].

### 3) Online Collaborative Research

As we saw above, informal blogging sites can be useful sources of advice on experimental methods. While the primary chemical literature remains the largest source of this kind of information, websites have the advantage that users may add or edit the information collaboratively. Organic Syntheses hosts open access, rigorously checked procedures [36], while Synthetic Pages is a website that enables informal user feedback [37]. Open source protocols sites in the life sciences are also available [38].

Finally, several organisations have developed on the web recently that are looking into large, self-contained problems in chemistry, where profit-driven research has not delivered. UsefulChem posts the raw data on approaches to synthetic targets of interest, which currently include drug candidates for malaria [39]. The possibilities of web-based collaboration in chemistry are clear here, in that scans of spectra and TLC plates, as well as video footage of reactions in progress, make it very simple for readers to contribute to the science.

We have recently started an open source collaborative group in biomedical research called the Synaptic Leap [40-42]. The organisation currently focusses on neglected tropical diseases, such as malaria, schistosomiasis and tuberculosis, and the site itself grew from the Tropical Diseases Initiative [43]. The aim is to coordinate wide-ranging research projects in chemistry, biology and informatics. For example, a "gene wiki" concept is currently being explored as a way for the community to discuss and prioritise genes and proteins requiring further study. A current chemistry project on the site is the development of an enantioselective synthesis of the main drug used for the treatment of schistosomiasis, praziquantel [PubChem 4891] [44]. The latter project is a perfect example of where open source can really deliver. The iterative improvement of the route to a drug that is of great importance to underdeveloped countries is of little interest to for-profit companies, but neither is it a priority for academia. We see open source collaboration as the only way to make research challenges like this tractable. Further, open source research communities could have great impact across drug discovery more generally as part of collaborations with more traditional big-pharma drug discovery programs [45].

## Conclusion

Open access resources and open source collaborations are emerging in the chemical sciences at a high rate, driven in essence by enormous recent advances in communication technologies. There are clear benefits to such resources as mechanisms for accelerating scientific research. Both succeed in proportion to how much we become involved.
